# Effect of patient’s age on the profitability of inpatient cardiac catheterization: a contribution margin analysis of frequently performed procedures over a 5-year period

**DOI:** 10.1186/s12913-017-1999-4

**Published:** 2017-01-18

**Authors:** Gunnar Plehn, Thomas Butz, Petra Maagh, Axel Meissner

**Affiliations:** 1 Department of Cardiology, Johanniter-Hospital Duisburg Rheinhausen, Kreuzacker 1-7, 47228 Duisburg, Germany; 2 Ruhr-University of Bochum, Universitätsstrasse 150, 44801 Bochum, Germany; 3 Department of Cardiology, Catholic Hospital Oberhausen, Wilhelmstrasse 34, 46145 Oberhausen, Germany; 4 Department of Cardiology, Cologne-Merheim-Hospital, Ostmerheimer Strasse 200, 51109 Cologne, Germany; 5 University of Witten/Herdecke, Alfred-Herrhausen-Strasse 50, 58448 Witten, Germany

**Keywords:** Contribution margin analysis, Economic evaluation, Procedural costs, Material expenditure, Young versus old

## Abstract

**Background:**

Due to a continuing age shift in the German society hospital providers are concerned about the additional costs associated with the treatment of elderly patients. It is not clear if cardiac catheterization in aged patients leads to higher resource utilization and if DRG-revenues do compensate for this factor.

**Methods:**

Procedure-related and administrative data of all patients who underwent cardiac catheterization at a tertiary heart center between 2007 and 2011 were collected and analyzed. Then a profitability analysis was performed by comparing the case related variable costs with the Diagnosis-related group (DRG) per case revenues. A particular emphasis was placed on a comparative analysis of identical clusters of procedures.

**Results:**

The most frequently performed catheterization procedure (*n* = 1800) was associated with significantly higher material expenditure in very old patients (178 ± 48 €) than in old (171 ± 28; *p* = 0.001) and young patients (172 ± 39; *p* = 0.046). Furthermore, radiation time and the length of hospital stay were increased in very old patients (3.5 ± 3.8 min and 6.2 ± 4.8 days) compared to old (2.7 ± 2.8 min and 4.6 ± 3.8 days; *p* < 0.001) and young patients (2.5 ± 2.5 min and 4.5 ± 3.9 days; *p* < 0.001). Due to higher DRG revenues very old patients achieved higher absolute contribution margins (2065 ± 1033 €) than old (1804 ± 1902 €; *p* < 0.001) and young patients (1771 ± 902 €; *p* < 0.001). However, the contribution margins per day were significantly smaller (440 ± 226 €) than those in old (488 ± 234 €; *p* = 0.001) and young patients (484 ± 206 €; *p* = 0.001).

**Conclusions:**

Catheterization of very old patients is related to lower contribution margins per day despite higher material and time expenditures. Since efforts to reduce the length of hospital stay of these patients are limited, this may result in a competitive disadvantage of hospitals which are more affected by the demographic change.

**Electronic supplementary material:**

The online version of this article (doi:10.1186/s12913-017-1999-4) contains supplementary material, which is available to authorized users.

## Background

The demographic aging of the German population is a historically grown irreversible process [1]. Thus, German hospitals have thus the task to secure the in-patient care of an increasing number of patients with typical age-related diseases. With respect to cardiovascular diseases, it is expected that healthcare providers will face a significant increase in patient numbers and in particular in old patients during the next two decades [2–4].

Healthcare providers are concerned about the necessary but cost-intensive concentration on the aging patient population. Multi-morbidity, a delayed recovery period and dementia are frequent issues associated with the elderly and require additional effort with respect to assistance and care. At the same time, the length of in-patient care will be prolonged [5]. Since German diagnosis-related groups (G-DRG) provide aggressive economic incentives to reduce the length of the in-patient stay as a flat-rate reimbursement system, and individual resource consumption is only considered to a limited extent, a systematic underfunding can be expected regarding this patient group [6, 7].

Our study targets the question whether the profitability of typical cardiac catheterization procedures differs according to patients’ age. Traditional costing systems do not distinguish between variable and fixed costs. As a consequence time and material expenses are not appropriately allocated to specific procedures providing an unsafe basis for strategic decision making. Contribution margin (CM) analysis is the preferred financial analysis tool in situations where the profitability of different patient groups has to be compared within an established service line [8, 9]. Knowledge of the contribution margin is considered as an essential step in order to identify those patient types, which contribute most to the coverage of a hospital’s unavoidable fixed cost burden and thus profitability [10].

To accomplish this task the procedure-related and administrative data of all patients who underwent cardiac catheterization at a tertiary heart center between 2007 and 2011 were collected and analyzed. To ensure the comparison of identical processes at the cardiac catheterization laboratory level the three most common clusters of procedures according the International Classification of Procedures in Medicine (ICPM) were chosen for subsequent age-related analyses.

## Methods

### Data collection and subgroup definition

In a first step all patients undergoing cardiac catheterization at a German University Medical Center from January 1, 2007 to December 31, 2011 were identified (*n* = 11786). Baseline and procedural data of these patients were derived from the cardiac catheterization database Metek (Metek, 52159 Roetgen, Germany). In particular, all materials utilized during the procedure were collected from the Metek database. The software provides a list of supply costs including catheters and other disposable equipment, radiographic contrast medium and medication. In a subsequent analytic step each material position was substituted by their corresponding Euro value, which has been generated from the purchase list of the material storage data base. In a further step the resulting database was combined with the hospitals’ information system (Clinicom CareCenter, Siemens) in a case by case manner. The final database therefore comprised procedural as well as administrative data necessary for coding within the German Diagnosis Related Groups (DRG) system.

The DRG payment each hospital receives is proportional to a relative cost weight which reflects the complexity of treatment and the relative costs of one DRG to another. The effective relative cost weight includes additional fees or reductions related to long- or short-stay patients.

Patients who had multiple catheterization procedures during one hospital stay and cases where patients received a combination of coronary and electrophysiological procedures during one visit were excluded. Furthermore, patients undergoing artificial respiration were excluded as well as those who received pacemaker therapy or any relevant procedure from other specialties (e.g. surgery or endoscopy). In sum, *n* = 2868 patients were excluded for these reasons. Furthermore, of the remaining patients those with incomplete data-sets were not included (*n* = 1588). All procedures were done by one of five interventional cardiologists with high experience levels. The final database included *n* = 7330 subjects.

To ensure that only identical catheterization procedures were chosen for age-related analyses ICPM codes were used to identify the three most frequently performed clusters of procedures (P1, P2 and P3). Procedure codes are a subtype of medical classification used to identify diagnostic or therapeutic procedures. Typical cardiac catheterization procedures comprise several ICPM codes forming a cluster.

Age-related subgroups were defined by dividing the entire database (*n* = 7330), as well as P1 (*n* = 1800), P2 (*n* = 446) and P3 (*n* = 409) into age tertiles. The results were further validated using an alternative subgroup definition by decade of age to determine age-related differences (Fig. 2).

### Contribution margin analysis

Our concept of a one-step contribution margin analysis implies the following: The assumed capacities in the area of inpatient care (e.g. room nursing costs, catering and overhead costs) are considered as organizational prerequisite for the value creating process within the catheterization laboratory [6, 11]. Accordingly, these costs are accordingly added to structure or fixed costs. Costs, which are procedure-related such as catheter equipment (material costs) or related to staff, cleaning or maintenance of the catheterization laboratories are considered as variable costs. The contribution margin amounts were analyzed on a case-by-case basis.

Contribution margin calculation included the following cost positions

- EMA = individual expenses for material and medication (per patient)
- EC = expenses for cleaning (10 hours per day on 250 days ≙ 22.500 € )
- EM = expenses for maintenance of two cath labs (≙ 60.000 €)
- EP = cath lab physicians costs (109 € per examination hour)
- EN = cath lab nursing costs (9 full-time employees, two part-time employees ≙ 185.013 € per year)

Expenses for cleaning, cath lab maintenance, nursing and physician costs were allocated to one hour of cath lab examination time (total cath lab expenses per hour = ECL _per hour_) on a 5 year basis. 5-year cumulative examination time amounted to 4.950 h.

$$ \begin{array}{l}\mathrm{E}\mathrm{C}{\mathrm{L}}_{\mathrm{per}\ \mathrm{hour}}=\left(5\times \left(\mathrm{E}\mathrm{C}+\mathrm{E}\mathrm{M}+\mathrm{E}\mathrm{N}\right)\right)+\left(\mathrm{E}\mathrm{P}\times 4.950\right)/4.950\Big)=379{\textsf{C}\hspace{-1.7ex}{=}} \\ {}\mathrm{Variable}\ \mathrm{costs}\ \mathrm{per}\ \mathrm{patient}=\mathrm{E}\mathrm{M}\mathrm{A}+\mathrm{examination}\ \mathrm{time}\ \mathrm{per}\ \mathrm{patient}\times 379{\textsf{C}\hspace{-1.7ex}{=}} \end{array} $$

The contribution margin for each case was calculated as the difference between DRG-revenue and variable costs [12]. Since the DRG system (as a per-case flat rate system) provides strong financial incentives to reduce the length of hospital stay, economic performance can be better evaluated on a per-day basis. Therefore, relative DRG-revenues (DRG-revenue per day) and relative contribution margins (CM per day) were introduced.

The study was approved by the Research Ethics Committee of Ruhr-University of Bochum (register number 3945–11). All participants gave their consent to take part.

## Statistics

To compare group means with respect to systematic differences, an analysis of variance (ANOVA) was performed. When variations between the means were found, post-hoc tests were performed to verify, which of both groups specifically differ. Before running post-hoc analysis, the relevant factors were examined with respect to homogeneity (Levene test). Depending on the results either the Bonferroni test (variance homogeneity) or the Tamhane T2 test (variance inhomogeneity) was applied. A multivariable stepwise regression model was used to describe the relationship between key indicators of resource consumption and profitability as material costs, variable costs, contribution margins per day and length of hospital stay (with each of them considered as the dependent variable) and a set of independent variables which demonstrated a significant association in preliminary univariate analysis. These variables comprised demographic attributes as age and gender, DRG relative weights (which drive DRG prices) and parameters of procedural resource consumption. Only parameters which were mathematically related were excluded from the multivariable regression model to avoid tautological relationships. Those models with the highest R2 were chosen. All analyses were performed using the software package SPSS for Windows 12.0.1 (SPSS, Chicago, IL).

## Results

### Age distribution of cardiac catheterization patients and age development within a 5 year period

**Fig. 1:**
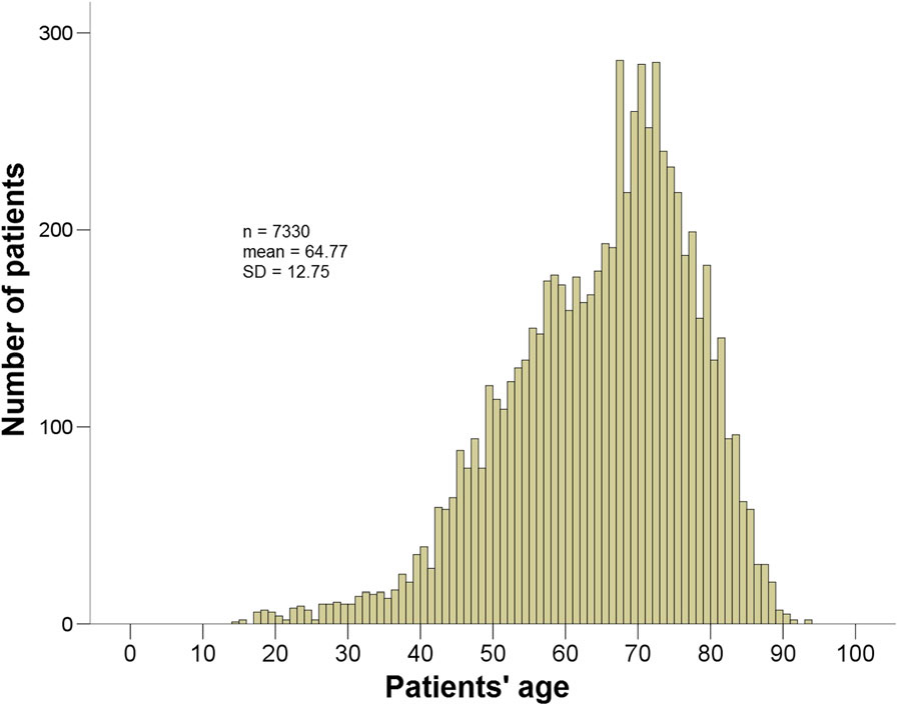
Age distribution of invasive cases treated in the period 2007–2011. Shape analysis shows a clear asymmetry with a right shift of the age peak. The highest case numbers per age group can be found in the groups with patients in their 70s and 80s

The mean age of the patient examined in the period 2007–2011 was 64.8 years. In considering the distribution of the case numbers with respect to the different age groups, a typical aging curve can be derived (Fig. 1). Shape analysis shows a clear asymmetry with a right shift of the age peak. The highest case numbers per age group can be found in the groups with patients in their 70s and 80s.

Variance analysis shows significant differences of the group means between the years 2007–2011. When the years 2007 and 2011 are directly compared, a significant increase of the mean age from 64.2 to 65.6 years was found (*p* = 0.009). Furthermore, a significant correlation between patients age and the chronology of their exams (continuous case numbers) during the study period 2007–2011 was found (r^2^ = 0.002; *p* < 0.001).

### Patients’ Age, costs and revenues: characteristics of the total population for the period 2007–2011

Based on the pooled databases of all heart catheter exams in the period 2007–2011 age tertiles were generated (A = young, B = old, C = very old; Table 1).

**Table 1:** Age-dependent treatment parameters of the total population with invasive treatments in the period 2007–2011

	Young	Old	Very old	*F*	*P-value*
	*n* = 2443	*n* = 2444	*n* = 2443		
	50 ± 9 years	67 ± 3 years	78 ± 4 years		
DRG revenue (Euro)	3403 ± 7016	3718 ± 4609	4292 ± 5020	17	<0.001
DRG revenue per day (Euro)	774 ± 511	758 ± 542	703 ± 542	13	<0.001
Contribution margin (Euro)	1824 ± 1124	2032 ± 1612	2436 ± 1878	96	<0.001
Contribution margin per day (Euro/d)	489 ± 299	488 ± 313	465 ± 315	4	0.01
Relative DRG weight	1.2 ± 1.9	1.3 ± 1.6	1.5 ± 1.7	21	<0.001
Length of hospital stay (d)	5.4 ± 6.4	6.4 ± 6.7	8.2 ± 8.0	107	<0.001
Fluoroscopy time (min)	7.2 ± 9.3	74 ± 8.6	7.9 ± 7.7	-	ns
Material expenditure (Euro)	519 ± 678	470 ± 575	435 ± 488	14	<0.001

*DRG* Diagnosis Related Groups

We were able to show that the average examination of very old patients causes lower material costs than of young patients. The exam’s duration was comparable with the other age groups. Moreover, higher relative case weights were noticeable as well as higher absolute DRG revenues. However, the length of hospital stay (LOS) was however clearly longer than the stay predetermined by the DRG. This effect was so strong that the daily DRG-revenues and daily contribution margins were clearly lower than in the other age groups. It is important to recognize that this level of analysis reflects a “procedure-unadjusted” comparison. Due to age-dependent differences of diagnoses and treatments the spectrum of procedures performed in different age groups probably differs.

### Effect of patients age on resource consumption and revenues

The three most frequent procedure clusters at the heart catheter lab refer to 36.2% of all treated cases. With decreasing frequency, they are diagnostic heart catheter exams with closure device (P1 = ICPM cluster 1–275.2, 1–279.0, 8–83b.c), diagnostic heart catheter exams without closure device (P2 = ICPM cluster 1–275.2, 1–279.0) and the combined diagnostic left and right heart catheterization with closure device (P3 = ICPM cluster 1–273.1, 1–275.3, 1–279.0, 8-83b.c).

Table 2 shows that the most frequent procedure P1 (*n* = 1,800) was clearly associated with age-dependent differences in resource consumption. The average material costs per exam in the case of very old patients about EUR 6–7 higher as compared to old and young patients. In addition, fluoroscopy was much more used and average examination times were longer in these patients. The mean LOS was about 1.5 days longer than in both comparison groups.

**Table 2:** Parameters of the most frequent procedures (P1-P3)

Procedure P1 Diagnostic catheter with closure device	Young	Old	Very old	*F*	*P-value*
	*n* = 600	*n* = 600	*n* = 600		
	48 ± 7 years	63 ± 7 years	76 ± 5 years		
DRG revenue (Euro)	2077 ± 917	2122 ± 1024	2398 ± 1028	18	<0.001
DRG revenue per day (Euro)	592 ± 253	600 ± 276	535 ± 278	11	<0.001
Contribution margin (Euro)	1771 ± 922	1804 ± 1029	2065 ± 1033	16	<0.001
Contribution margin per day (Euro/d)	484 ± 206	488 ± 234	440 ± 226	9	<0.001
Relative DRG weight	0.73 ± 0.27	0.77 ± 0.37	0.85 ± 0.34	19	<0.001
LOS (d)	4.5 ± 3.9	4.6 ± 3.8	6.2 ± 4.8	33	<0.001
Fluoroscopy time (min)	2.5 ± 2.5	2.7 ± 2.8	3.5 ± 3.8	19	<0.001
Material expenditure (Euro)	172 ± 39	171 ± 28	178 ± 48	4.6	0.01
Procedure P2 Diagnostic catheter without closure device	Young	Old	Very old	*F*	*P-value*
	*n* = 149	*n* = 149	*n* = 148		
	50 ± 9 years	62 ± 4 years	76 ± 5 years		
DRG revenue (Euro)	2079 ± 798	1994 ± 1029	2383 ± 1193	6.3	0.003
DRG revenue per day (Euro)	579 ± 243	586 ± 268	497 ± 261	6.0	0.004
Contribution margin (Euro)	1801 ± 812	1695 ± 1020	2079 ± 1120	5.7	0.004
Contribution margin per day (Euro/d)	482 ± 200	472 ± 223	419 ± 211	3.9	0.02
Relative weight	0.74 ± 0.26	0.73 ± 0.33	0.81 ± 0.33	5.6	ns
Length of hospital stay (d)	4.5 ± 3.0	4.5 ± 4.1	7.0 ± 6.3	14	<0.001
Fluoroscopy time (min)	3.7 ± 4.0	5.6 ± 6.6	5.2 ± 5.1	4.4	0.01
Material expenditure (Euro)	104 ± 34	109 ± 39	122 ± 56	836	0.002
Procedure P3 Combined diagnostic left and right heart catheter with closure device	Young	Old	Very old	*F*	*P-value*
	*n* = 136	*n* = 136	*n* = 137		
	52 ± 8 years	67 ± 3 years	77 ± 4 years		
DRG revenue (Euro)	2021 ± 697	2225 ± 1197	2681 ± 3008	4.2	0.01
DRG revenue per day (Euro)	604 ± 219	566 ± 208	516 ± 211	5.9	0.003
Contribution margin (Euro)	1646 ± 695	1827 ± 1182	2266 ± 3018	3.8	0.02
Contribution margin per day (Euro/d)	475 ± 180	444 ± 166	415 ± 172	4.3	0.02
Relative weight	0.73 ± 0.25	0.79 ± 0.38	0.94 ± 1.1	3.7	0.02
Length of hospital stay (d)	4.1 ± 3.1	5.1 ± 5.1	6.6 ± 6.2	8.7	<0.001
Fluoroscopy time (min)	3.9 ± 4.0	4.4 ± 5.2	4.6 ± 6.6	-	ns
Material expenditure (Euro)	219 ± 30	221 ± 34	238 ± 163	-	ns

*DRG* Diagnosis Related Groups

With respect to absolute DRG and contribution margin revenues higher amounts were achieved per single treatment-case. The very old patient is considered to be sicker and receives higher relative DRG cost weights which drive DRG revenues. Looking at the per day profitability a reversed situation became obvious. The higher absolute revenues were used up or reversed by an increased LOS of very old patients. Per treatment day, very old patients achieved smaller DRG revenues and this effect translated into smaller per day contribution margins. The results of the P1 analysis of invasive treatment cases were basically confirmed by the P2 and P3 analysis (Table 2). Across all procedure complexes and thus procedure-independently, the relative contribution margin was significantly lower in the case of very old patients compared to younger patients.

**Fig. 2:**
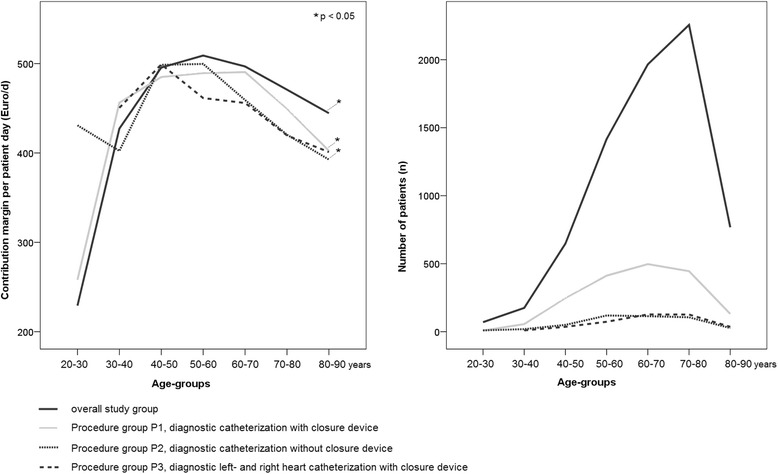
Distribution of contribution margin per day revenues across decades of age and corresponding number of patients. The contribution margin per day revenues of the total patient group and the P1-P3 subgroups form convex shaped curves. Their peaks indicate that the highest per day contribution margins can be realized in age-groups between 40 and 70 years. The right diagram shows that the decade groups over 60 years are the numerically most important groups

Because of the arbitrary nature of the categories used in our primary analysis an extensive sensitivity analysis was performed. By using an alternative subgroup definition by decades of age an analysis of variance confirmed significant age-related differences in per day contribution margins (with exception auf subgroup P3 with a *p* = 0.085; Fig. 2). Multivariable regression modeling demonstrated that key measures of resource consumption and profitability as LOS or contribution margin per day amounts were inconsistently explained by DRG-relative cost weights alone. In most evaluated scenarios resource consumption was best explained by models including procedural data and patients’ age as covariates (see Additional file 1).

## Discussion

Our findings suggest that age has an independent impact on resource utilization in common left heart catheterization procedures as coronary angiography and that the cost-relevant effect of age is not adequately reflected by the German DRG-system. Catheterization procedures are characterized by a high percentage of manual activity and high material expenses. Given the high proportion of variable costs, an important strength of our cost analysis is that we were able to assess variable costs across a large number of identical ICPM clusters. Unlike those studies on cost effectiveness that rely on average or aggregate cost [13], analyses based on patient-level data are considered to be more robust and insightful [14–16]. Irrespective of the following discussion on a DRG-level our findings demonstrate that coronary angiography which is one of the most commonly performed procedures worldwide, is associated with higher material expenditure and radiation time when performed in elderly patients. From a clinical point of view older age may be associated with more complex vascular morphology, vessel elongation and tortuosity leading to longer procedures and fluoroscopy times [17, 18]. Access site complications occurred more frequently in patients who were of older age [19].

It has been suggested that an advanced age is strongly associated with an increased burden of comorbidity and a higher proportion of patients with comorbidities [20]. However, the effects of age and comorbidity are not synonymous. Health status and clinical outcomes are typically influenced by both factors: age and comorbidities. Disease entities as hemodialysis and sepsis illustrate that an advanced age and a high comorbidity index are significant and independent predictors of mortality [21, 22]. Furthermore, age and comorbidities were found to be independent predictors of death and of major bleeding complications after percutaneous coronary intervention [23–25]. Similar observations were made from a health service perspective. Only few studies demonstrated an isolated effect of rather age or comorbidities on fiscal outcome [26–28]. The majority of evidence suggests that age and comorbidities are independently related to resource utilization including costs, length of hospital stay and physician visits [22, 29, 30]. For instance the length of hospital stay which has frequently been used as a proxy of hospital costs [5] was found to be independently related to age and comorbidities in patients with sepsis and trauma [21, 31]. Older age at entry and comorbidities increased the costs of short and long-term inpatient care of elderly hypertensive patients [32]. In addition, age and the Charlson-comorbidity Index were shown to be independent predictors of increased hospitalization costs for myocardial infarction patients treated with PCI [33]. The awareness of the importance of age as an independent predictor of clinical and fiscal outcomes has further influenced the design and interpretation of comorbidity scoring systems. A composite age/comorbidity score which accurately accounts for the impact of age and comorbidity was proposed as a tool for making treatment decisions and estimating outcomes in allogenic hematopoietic cell transplantation [34]. The Davies score is a commonly used comorbidity index which is specifically designed to be used in conjunction with age as an independent covariate [35].

Payment systems based on DRGs have been widely adopted internationally and provide a per-case flat rate payment [36]. Every DRG is linked to a relative cost weight which reflects the amount of resources requirements an average patient in that DRG is expected to consume. Ideally, relative weights should be in parallel with the hospital costs for each DRG and thus perfectly explain differences in patients’ diagnoses, comorbidities and procedures [37, 38]. The amount of DRG-revenue which exceeds variable costs (= contribution margin) should reflect those cost components which can be allocated to non-procedural patient characteristics as differences in diagnoses, age and comorbidities.

From a hospitals perspective, this proportion of revenues serves to cover fixed costs as ward nursing, ward medicals, social service, catering as well as laboratory and overhead costs. Our findings indicate that DRG-relative weight indices incompletely explain variations in resource consumption among patients undergoing identical cardiac catheterization procedures. Although DRG-relative weights and thus DRG-revenues were much higher in very old patients the gain in revenues did not compensate for an increase in resource utilization and in particular for a prolonged length of hospital stay in these patients. Several studies indicated that there is a strong correlation between age and length of hospital stay [39]. The patients who stay in hospital for a very long time are usually those that consume the largest amount of hospital resources [5]. As a consequence discharges of old patients are undervalued compared to younger patients and hospitals that tend to serve more old patients are underpaid compared to those caring for younger patients. The unified medical reimbursement of the DRG payment system implies that the risk of particular complex cases is rendered by the DRG to the hospitals [40]. This regulation seems to be acceptable as long as the outliers of the costs are exceptions. However, if the exception becomes the rule, a significant financial risk can arise and can threat the relevant providers [40–42]. These inaccuracies of payment may provoke behavioral changes in hospital recruitment or discharge strategies which in turn may have a detrimental effect on the care of elderly patients.

Concerns about the appropriateness of resource allocation within the German DRG system have been previously raised. Geissler et al. analyzed administrative data of more than 50 thousand cases and revealed that DRG payments poorly reflect the true resource consumption of patients [6]. In particular, simple patient data as the number of diagnoses or procedures showed a closer relationship to resource consumption than DRG revenues. The findings were in marked contrast to a previously reported analysis on a European-level by the same authors which demonstrated that several national DRG systems perform equally or better than simple patient data [43]. The similar question was addressed by a study on breast cancer surgery reimbursements in 10 European countries. In 7 out of 10 national samples, including Germany, routine patient data performed at least as well as the national DRG systems in accounting for patient-level variation in resource consumption. The study further revealed that patients’ age (71 years and older) is a significant determinant of resource consumption that could be favorably integrated into DRG-algorithms to increase resource homogeneity [7].

To serve as a basis for a fair hospital payment DRG systems need to be able to define patients resource consumption as sufficient as possible. Our findings indicate that age has to be considered as a significant determinant of resource consumption in patients undergoing cardiac catheterization. In case of invasive cardiac diagnostic procedures an age split has already been established within the German-DRG system for children under 15 years. We suggest that the introduction of a adult age split may similarly help to improve the reimbursement allocation in elderly patients and to better meet the demands of an aging society. Moreover, there is growing evidence that age-adjustment can improve DRG case homogeneity in many other medical fields [7, 43, 44]. Simple routine patient data were shown to adequately or better reflect resource consumption than highly complex DRG algorithm that distinguish many procedural subgroups [7, 43]. Such adjustments may pave the way for a more patient-oriented and simplified reimbursement system.

## Limitations

The study represents a single center experience and generalizability may thus be limited. Processes show a considerable variation across cardiac catheterization laboratories and material costs may differ depending on hospital contracts. However, the additional efforts associated with catheterization of aged patients are a common issue relevant to many medical users.

The contribution margin concept does not include fixed costs such as room nursing costs, laboratory costs and overhead costs. Its strength lies in a comprehensive reflection of those costs (variable costs) which can be directly influenced by the catheterization team.

## Conclusions

Catheterization in elderly patients is associated with an increased utilization of hospital resources. Variable costs are higher and per-day DRG-revenues are reduced due to a longer length of hospital stay. Both factors lead to lower per-day contribution margins and thus to a lower profitability of these cases. Efforts to understand and control higher material and time expenses in elderly patients may help to develop age-adapted catheterization concepts in order to reduce variable costs. Our findings further support the need for a refinement of DRG algorithms. Incorporation of simple variables such as patients’ age may help to better account for patient-level resource consumption and to meet the upcoming demographic challenge.

## Additional files

Additional file 1: Supplemental data sheet with extended version of Tables S1 and S2 from the manuscript including post-hoc analysis between age groups; extended version of Figure S2 including raw data and multivariable regression analysis of independent predictors of resource utilization and profitability. (DOCX 146 kb)
Additional file 2: Original anonymous data set. (XLS 9209 kb)

## Abbreviation

ALOS: Predetermined average length of hospital stay by DRG
cGy: Centigray
CM: Contribution margin
DRG: Diagnosis related groups
ICPM: International classification of procedures in medicine
LOS: Length of Hospital stay
OPS: German procedure classification code
P1-P3: Procedure cluster 1–3
