# Supplementary material for: Effect of patient’s age on the profitability of inpatient cardiac catheterization: a contribution margin analysis of frequently performed procedures over a 5-year period
Source: BMC Health Serv Res. 2017 Jan 18;17:49. doi: 10.1186/s12913-017-1999-4 (PMC5241940; doi:10.1186/s12913-017-1999-4)
Supplement: Additional file 1: — Supplemental data sheet with extended version of Tables S1 and S2 from the manuscript including post-hoc analysis between age groups; extended version of Figure S2 including raw data and multivariable regression analysis of independent predictors of resource utilization and profitability. (DOCX 146 kb) [file 12913_2017_1999_MOESM1_ESM.docx]

### Effect of patient’s age on the profitability of inpatient cardiac catheterization: a contribution margin analysis of frequently performed procedures over a 5-year period

Gunnar Plehn^1^, Thomas Butz^2^, Petra Maagh^3^ and Axel Meissner^3^

**Supplemental data sheet**

1. Extended version of Table 1 and 2 including post-hoc analysis between age groups

2. Extended version of Figure 2 including raw data

3. Independent predictors of resource utilization and profitability in multivariable regression analysis (Table 3)

Table 1: *Age-dependent treatment parameters of the total population with invasive procedures in the period 2007-2011.*

|  | **young (A)**  **n = 2443**  **50±9 years** | | | **old (B)**  **n = 2444**  **67±3 years** | | | **very old (C)**  **n = 2443**  **78±4 years** | | | ***F*** | ***P-value*** | ***A vsB*** | ***B vs C*** | ***A vs C*** |
| --- | --- | --- | --- | --- | --- | --- | --- | --- | --- | --- | --- | --- | --- | --- |
| **DRG revenue (Euro)** | 3403 | ± | 7016 | 3718 | ± | 4609 | 4292 | ± | 5020 | 17 | <0.001 |  | x | x |
| **DRG revenue per day (Euro)** | 774 | ± | 511 | 758 | ± | 542 | 703 | ± | 542 | 13 | <0.001 |  | x | x |
| **relative weight** | 1.2 | ± | 1.9 | 1.3 | ± | 1.6 | 1.5 | ± | 1.7 | 21 | <0.001 |  | x | x |
| **effective relative weight** | 1.2 | ± | 2.4 | 1.3 | ± | 1.5 | 1.5 | ± | 1.8 | 17 | <0.001 |  | x | x |
| **LOS (d)** | 5.4 | ± | 6.4 | 6.4 | ± | 6.7 | 8.2 | ± | 8.0 | 107 | <0.001 | x | x | x |
| **ALOS – LOS (d)** | -0.05 | ± | 4.6 | -0.6 | ± | 5.2 | -1.5 | ± | 6.3 | 45 | <0.001 | x | x | x |
| **preprocedural days** | 2.2 | ± | 4.6 | 2.5 | ± | 4.1 | 3.0 | ± | 4.6 | 25 | <0.001 |  | x | x |
| **examination time (min)** | 41.3 | ± | 38.7 | 40.6 | ± | 35.9 | 40.5 | ± | 30.7 | 0.48 | ns |  |  |  |
| **fluoroscopy time (min)** | 7.2 | ± | 9.3 | 74 | ± | 8.6 | 7.9 | ± | 7.7 | 1.5 | ns |  |  |  |
| **radiation dose (cGy)** | 4617 | ± | 7471 | 5053 | ± | 7021 | 5271 | ± | 8611 | 4.9 | 0.008 |  |  | x |
| **material expenditure (Euro)** | 519 | ± | 678 | 470 | ± | 575 | 435 | ± | 488 | 14 | <0.001 | x |  | x |
| **CM (Euro)** | 1824 | ± | 1124 | 2032 | ± | 1612 | 2436 | ± | 1878 | 96 | <0.001 | x | x | x |
| **relative CM (Euro/d)** | 489 | ± | 299 | 488 | ± | 313 | 465 | ± | 315 | 4 | 0.01 |  | x | x |

A,B,C = age tertiles; a = years; cGy = centigray; DRG = Diagnosis Related Groups; ALOS = predetermined average length of hospital stay by DRG; LOS = length of hospital stay; x = significance in post hoc analysis

Table 2: *Parameters of the most frequent procedures (P1-P3).*

| procedure P1 diagnostic catheter with closure device | | **A**  **n = 600**  **48±7 years** | | | | | | **B**  **n = 600**  **63±7 years** | | | | | | **C**  **n = 600**  **76±5 years** | | | | | | ***F*** | | ***P-value*** | | ***A vs B*** | | ***B vs C*** | | ***A vs C*** | |
| --- | --- | --- | --- | --- | --- | --- | --- | --- | --- | --- | --- | --- | --- | --- | --- | --- | --- | --- | --- | --- | --- | --- | --- | --- | --- | --- | --- | --- | --- |
| **DRG revenue (Euro)** | | 2077 | | ± | | 917 | | 2122 | | ± | | 1024 | | 2398 | | ± | | 1028 | | 18 | | <0.001 | |  | | x | | x | |
| **DRG revenue per day (Euro)** | | 592 | | ± | | 253 | | 600 | | ± | | 276 | | 535 | | ± | | 278 | | 11 | | <0.001 | |  | | x | | x | |
| **relative DRG weight** | | 0.73 | | ± | | 0.27 | | 0.77 | | ± | | 0.37 | | 0.85 | | ± | | 0.34 | | 19 | | <0.001 | | x | | x | | x | |
| **effective relative DRG weight** | | 0.73 | | ± | | 0.32 | | 0.75 | | ± | | 0.36 | | 0.85 | | ± | | 0.38 | | 18 | | <0.001 | |  | | x | | x | |
| **LOS (d)** | | 4.5 | | ± | | 3.9 | | 4.6 | | ± | | 3.8 | | 6.2 | | ± | | 4.8 | | 33 | | <0.001 | |  | | x | | x | |
| **ALOS – LOS (d)** | | 0.36 | | ± | | 3.4 | | 0.46 | | ± | | 3.0 | | -0.55 | | ± | | 3.9 | | 15 | | <0.001 | | x | | x | | x | |
| **preprocedural days l** | | 2.1 | | ± | | 2.1 | | 2.1 | | ± | | 2.3 | | 2.75 | | ± | | 3.1 | | 12 | | <0.001 | |  | | x | | x | |
| **examination time (min)** | | 21.1 | | ± | | 13.0 | | 23.3 | | ± | | 23.7 | | 24.6 | | ± | | 20.6 | | 4.9 | | 0.007 | |  | |  | | x | |
| **fluoroscopy time (min)** | | 2.5 | | ± | | 2.5 | | 2.7 | | ± | | 2.8 | | 3.5 | | ± | | 3.8 | | 19 | | <0.001 | |  | | x | | x | |
| **radiation dose (cGy)** | | 2842 | | ± | | 3636 | | 2946 | | ± | | 3551 | | 3043 | | ± | | 2755 | |  | | ns | |  | |  | |  | |
| **material expenditure (Euro)** | | 172 | | ± | | 39 | | 171 | | ± | | 28 | | 178 | | ± | | 48 | | 4.6 | | 0.01 | |  | | x | | x | |
| **CM (Euro)** | | 1771 | | ± | | 922 | | 1804 | | ± | | 1029 | | 2065 | | ± | | 1033 | | 16 | | <0.001 | |  | | x | | x | |
| **CMd (Euro/d)** | | 484 | | ± | | 206 | | 488 | | ± | | 234 | | 440 | | ± | | 226 | | 9 | | <0.001 | |  | | x | | x | |
| procedure P2 diagnostic catheter without closure device | **A**  **n = 149**  **50±9 years** | | | | | | **B**  **n = 149**  **62±4 years** | | | | | | **C**  **n = 148**  **76±5 years** | | | | | | ***F*** | | ***P-value*** | | ***A vs B*** | | ***B vs C*** | | ***A vs C*** | |  |
| **DRG revenue (Euro)** | 2079 | | ± | | 798 | | 1994 | | ± | | 1029 | | 2383 | | ± | | 1193 | | 6.3 | | 0.003 | |  | | x | | x | |  |
| **DRG revenue per day (Euro)** | 579 | | ± | | 243 | | 586 | | ± | | 268 | | 497 | | ± | | 261 | | 6.0 | | 0.004 | |  | | x | | x | |  |
| **relative DRG weight** | 0.74 | | ± | | 0.26 | | 0.73 | | ± | | 0.33 | | 0.81 | | ± | | 0.33 | | 5.6 | | ns | |  | |  | |  | |  |
| **effective relative DRG weight** | 0.73 | | ± | | 0.28 | | 0.71 | | ± | | 0.36 | | 0.84 | | ± | | 0.42 | | 5.8 | | 0.002 | |  | | x | | x | |  |
| **LOS (d)** | 4.5 | | ± | | 3.0 | | 4.5 | | ± | | 4.1 | | 7.0 | | ± | | 6.3 | | 14 | | <0.001 | |  | | x | | x | |  |
| **ALOS – LOS (d)** | 0.36 | | ± | | 0.23 | | 0.23 | | ± | | 3.0 | | -1.3 | | ± | | 5.3 | | 9.4 | | <0.001 | |  | | x | | x | |  |
| **preprocedural days l** | 2.4 | | ± | | 2.1 | | 2.5 | | ± | | 5.1 | | 3.1 | | ± | | 3.9 | |  | | ns | |  | |  | |  | |  |
| **examination time (min)** | 27 | | ± | | 18 | | 30 | | ± | | 18 | | 29 | | ± | | 16 | |  | | ns | |  | |  | |  | |  |
| **fluoroscopy time (min)** | 3.7 | | ± | | 4.0 | | 5.6 | | ± | | 6.6 | | 5.2 | | ± | | 5.1 | | 4.4 | | 0.01 | | x | |  | |  | |  |
| **radiation dose (cGy)** | 3212 | | ± | | 3441 | | 4658 | | ± | | 9853 | | 3587 | | ± | | 2499 | |  | | ns | |  | |  | |  | |  |
| **material expenditure (Euro)** | 104 | | ± | | 34 | | 109 | | ± | | 39 | | 122 | | ± | | 56 | | 836 | | 0.002 | |  | | x | | x | |  |
| **CM (Euro)** | 1801 | | ± | | 812 | | 1695 | | ± | | 1020 | | 2079 | | ± | | 1120 | | 5.7 | | 0.004 | |  | | x | |  | |  |
| **CMd (Euro/d)** | 482 | | ± | | 200 | | 472 | | ± | | 223 | | 419 | | ± | | 211 | | 3.9 | | 0.02 | |  | |  | | x | |  |
| procedure P3 diagnostic left- and right heart catheter with closure device | | **A**  **n = 136**  **52±8 years** | | | | | | **B**  **n = 136**  **67±3 years** | | | | | | **C**  **n = 137**  **77±4 years** | | | | | | ***F*** | | ***P-value*** | | ***A vs B*** | | ***B vs C*** | | ***A vs C*** |  |
| **DRG revenue (Euro)** | | 2021 | | ± | | 697 | | 2225 | | ± | | 1197 | | 2681 | | ± | | 3008 | | 4.2 | | 0.01 | |  | |  | | x |  |
| **DRG revenue per day (Euro)** | | 604 | | ± | | 219 | | 566 | | ± | | 208 | | 516 | | ± | | 211 | | 5.9 | | 0.003 | |  | |  | | x |  |
| **relative DRG weight** | | 0.73 | | ± | | 0.25 | | 0.79 | | ± | | 0.38 | | 0.94 | | ± | | 1.1 | | 3.7 | | 0.02 | |  | |  | | x |  |
| **effective relative DRG weight** | | 0.72 | | ± | | 0.25 | | 0.79 | | ± | | 0.43 | | 0.95 | | ± | | 1.1 | | 4.2 | | 0.02 | |  | |  | | x |  |
| **LOS (d)** | | 4.1 | | ± | | 3.1 | | 5.1 | | ± | | 5.1 | | 6.6 | | ± | | 6.2 | | 8.7 | | <0.001 | |  | | x | | x |  |
| **ALOS – LOS (d)** | | 0.96 | | ± | | 2.7 | | 0.46 | | ± | | 4.0 | | -0.23 | | ± | | 4.6 | | 3.2 | | 0.04 | |  | |  | | x |  |
| **preprocedural days l** | | 1.7 | | ± | | 2.0 | | 2.0 | | ± | | 2.5 | | 2.9 | | ± | | 3.8 | | 6.3 | | 0.002 | |  | | x | | x |  |
| **examination time (min)** | | 25 | | ± | | 15 | | 28 | | ± | | 23 | | 28 | | ± | | 23 | |  | | ns | |  | |  | |  |  |
| **fluoroscopy time (min)** | | 3.9 | | ± | | 4.0 | | 4.4 | | ± | | 5.2 | | 4.6 | | ± | | 6.6 | |  | | ns | |  | |  | |  |  |
| **radiation dose (cGy)** | | 3554 | | ± | | 2402 | | 3527 | | ± | | 2262 | | 3732 | | ± | | 2862 | |  | | ns | |  | |  | |  |  |
| **material expenditure (Euro)** | | 219 | | ± | | 30 | | 221 | | ± | | 34 | | 238 | | ± | | 163 | |  | | ns | |  | |  | |  |  |
| **CM (Euro)** | | 1646 | | ± | | 695 | | 1827 | | ± | | 1182 | | 2266 | | ± | | 3018 | | 3.8 | | 0.02 | |  | |  | | x |  |
| **CMd (Euro/d)** | | 475 | | ± | | 180 | | 444 | | ± | | 166 | | 415 | | ± | | 172 | | 4.3 | | 0.02 | |  | |  | | x |  |

A,B,C = age tertiles; a = years; cGy = centigray; CM = contribution margin; CMd = contribution margin per day; DRG = Diagnosis Related Groups; ALOS = predetermined average length of hospital stay by DRG; LOS = length of hospital stay

**Figure 2: *Distribution of contribution margin per day revenues across decades of age and corresponding number of patients.*** *The graphs illustrate that the contribution margin per day revenues of the total patient group and the P1-P3 subgroups form convex shaped curves. Their peaks indicate that the highest per day contribution margins can be realized in age-groups between 40 and 70 years. The decline in CM per day amounts towards young adults may be well allocated to lower service costs mainly due to a shorter length of hospital stay. In contrast, patients older than 70 years generate smaller amounts despite higher expected efforts. The right diagram shows that the decade groups over 60 years are the numerically most important groups.*


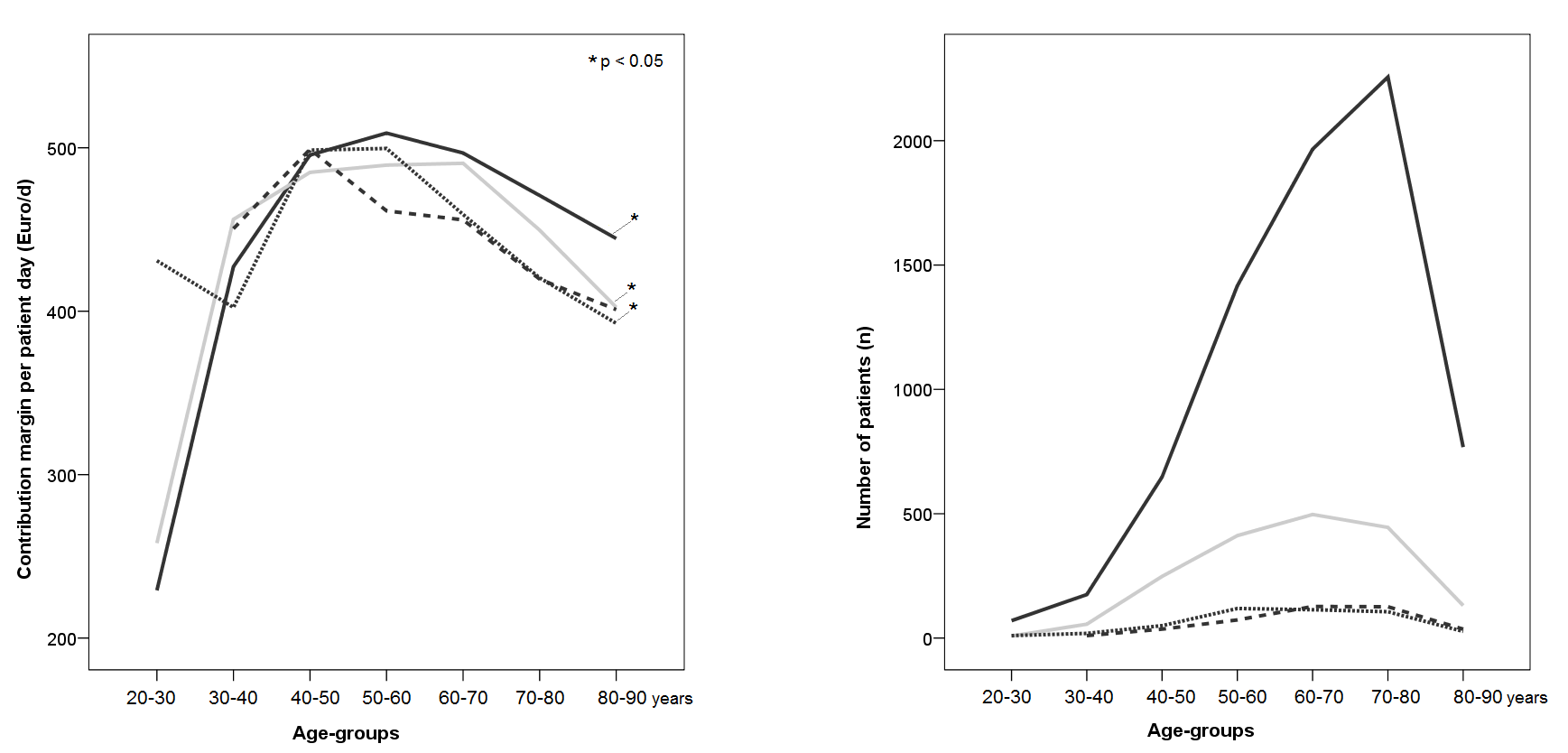


| **age-decade** | **20-30** | | **30-40** | | **40-50** | | **50-60** | | **60-70** | | **70-80** | | **80-90** | | **p** |
| --- | --- | --- | --- | --- | --- | --- | --- | --- | --- | --- | --- | --- | --- | --- | --- |
| P1 (n) | 8 |  | 56 |  | 248 |  | 412 |  | 497 |  | 445 |  | 131 |  |  |
| CMd | 258 | ±93 | 456 | ±93 | 485 | ±198 | 489 | ±212 | 490 | ±238 | 449 | ±235 | 402 | ±199 | < .001 |
| P2 (n) | 10 |  | 19 |  | 50 |  | 119 |  | 114 |  | 106 |  | 27 |  |  |
| CMd | 431 | ±204 | 402 | ±182 | 499 | ±195 | 500 | ±221 | 459 | ±201 | 420 | ±216 | 393 | ±227 | = .03 |
| P3 (n) |  |  | 10 |  | 36 |  | 73 |  | 127 |  | 126 |  | 36 |  |  |
| CMd |  |  | 451 | ±159 | 499 | ±234 | 461 | ±164 | 456 | ±161 | 420 | ±164 | 401 | ±193 | 0.085 |
| Total (n) | 70 |  | 175 |  | 648 |  | 1417 |  | 1966 |  | 2256 |  | 768 |  |  |
| CMd | 229 | ±240 | 427 | ±303 | 496 | ±274 | 509 | ±306 | 497 | ±318 | 471 | ±310 | 445 | ±312 | <.001 |

CMd = contribution margin per day; P1 = procedure cluster 1-275.2, 1-279.0, 8-83b.c (diagnostic catheter with closure device); P2 = procedure cluster 1-275.2, 1-279.0 (diagnostic catheter without closure device); P3 = procedure cluster 1-273.1, 1-275.3, 1-279.0, 8-83b.c (combined diagnostic left and right heart catheter with closure device) ; P1-P3 represent homogeneous cluster of procedures according to the ICPM code (International Classification of Procedures in Medicine)

**Table 3*.*** ***Independent predictors of resource consumption and profitability in multivariable regression analysis***

| **predictors** | **indicator**  **P1** (n = 1800) | | | | **indicator**  **P2** (n = 446) | | | | **indicator**  **P3** (n = 409) | | | | **indicator**  **overall** (n = 7330) | | | |
| --- | --- | --- | --- | --- | --- | --- | --- | --- | --- | --- | --- | --- | --- | --- | --- | --- |
|  | β (effect size) | | | | β (effect size) | | | | β (effect size) | | | | β (effect size) | | | |
|  | MC | VC | CMd | LOS | MC | VC | CMd | LOS | MC | VC | CMd | LOS | MC | VC | CMd | LOS |
| age |  | ,09** | -,05* | ,08** | ,11* |  |  | ,08* |  |  | -,10* | ,08* | -,06** | -,12** | ,03* | ,07** |
| gender | -.04* |  |  |  |  | -,11* |  |  |  |  |  |  |  | -,02* | -,02* | ,03** |
| material costs | t | t | -,05* |  |  |  |  |  | t | t | -,13* | ,11** | t | t | -,28** | -,19** |
| examination time | ,06* | t | -,17** |  |  |  | -,28** |  |  | t | -,25** |  | ,49** | t | -,18** |  |
| LOHS |  |  | t |  |  | ,13* | t |  | ,11* | ,26** |  |  | -,27** |  | -,88** |  |
| fluoroscopy time (min) | ,31** | t |  |  | ,25** |  |  |  | ,14* | t |  |  | ,07** | t |  |  |
| radiation dose (cGy) |  | t |  | ,06** |  |  |  | ,06* |  | t |  |  | ,08** | t |  |  |
| effective relative DRG weight |  |  |  | 1,34** |  |  | -,21** | 1,3** |  |  | 0,93* | 3,2** | ,28** |  | ,55** | 1,5** |
| relative DRG weight | .05* |  |  | -,71** | ,12* |  |  | -,58** |  | -,13* | 1,0** | -2,5** | ,10** | ,45** | ,21** | -,85** |

β, standardized regression coefficient; **P* < 0.05, ***P* < 0.001. t = tautological related variables not introduced into the regression model

MC = material costs; VC = variable costs; CMd = contribution margin per day; LOS = length of hospital stay; P1 = procedural cluster 1-275.2, 1-279.0, 8-83b.c (diagnostic catheter with closure device); P2 = procedural cluster 1-275.2, 1-279.0 (diagnostic catheter without closure device); P3 = procedural cluster 1-273.1, 1-275.3, 1-279.0, 8-83b.c (combined diagnostic left and right heart catheter with closure device) ; P1-P3 represent homogeneous cluster of procedures according to the ICPM code (International Classification of Procedures in Medicine)

*A multivariable stepwise regression model was used to describe the relationship between key indicators of resource consumption and profitability (with each of them considered as the dependent variable) and a set of independent variables which demonstrated a significant association in preliminary univariate analysis. The model demonstrates that key indicators of profitability and resource consumption as LOS or the contribution margin per day amounts were inconsistently explained by DRG-relative cost weights alone. In most evaluated scenarios resource consumption was best explained by models including procedural data and patients’ age as covariates. Within the largest homogenous group (P3) the contribution margin per day amounts were solely explained by age and procedural variables as material costs and examination time. Although their relative contributions were small, age and material costs were independently associated with the contribution margin per day amounts and LOS in the overall study group.*
